# Puerperal Infection

**Published:** 1904-06

**Authors:** 


### *SELECTIONS AND ABSTRACTS.*

---

### Puerperal Infection.*

Half a century has rolled away since that memorable Friday evening in Boston when our own Oliver Wendell Holmes read his epoch-making paper on "The Contagiousness of Puerperal Fever." Listerism, with its modifications, has robbed operative surgery of its terrible mortality; yet to-day motherhood is surrounded by the awful death-rate of nearly ten per cent., and of that mortality, more than seventy-five per cent. is due to *puerperal infection.* Surely this is a standing opprobrium to our boasted scientific advancement and acquisition at the opening of the twentieth century.

The pathology and etiology of puerperal infection is as thoroughly understood, and the methods of prophyalaxis as easy of application, as are those of any disease in our nomenclature.

Among the organisms causing puerperal infection may be mentioned (a) the streptococcus; (b) the staphylococcus; (c) the gonococcus; (d) the bacilus coli communis; (e) bacillus diphtheria; (f) the gas bacillus, or the bacillus of Welch; (g) the bacillus typhosus; (h) sapremia.

There is a very wide significance as to which or what germ or germs are found in a given case. It is very rare, indeed, that the streptococcus is found in the milder cases of infection, while it is well-nigh invariably found in the severer ones. In the milder cases, such germs as the *gonococcus*, *staphylococcus* and various other bacteria will be found. The streptococcic form of infection, therefore, will demand the closest attention and the most guarded prognosis.

If we would live up to the scientific light of to-day we will not neglect the bacteriological examination of the lochia. If we find the gonococcus we can heave a sigh of relief.

---

* Read by Edward T. Abrams, A.M., M.D., Dollar Bay, Mich., before the Houghton County Medical Society.

If the colon bacillus be discovered to be the cause, or if it be any of the ordinary putrefactive organisms, we may be spared much worry and apprehension; and yet it would be only prudent not to indulge in too much faith, hope and assurance from the bacteriological findings, for in some cases we may be led astray, and in not a few, perhaps a very large percentage, we shall have to do with a mixed infection. Let us not forget that all cases of puerperal infection do not carry with them the same grave prognosis. Thus it is we sometimes hear of puerperal fever being cured by small doses of phenacetine, or by nothing more energetic than the vaginal douche.

From an anatomico-pathological standpoint, we may divide puerperal infection into three forms, viz.: endometritis, metritis and parametritis.

Endometritis may be subdivided into *putrid* and *septic*. Putrid endometritis is, in the vast majority of cases, caused by a remaining portion of placenta or membrane in the uterine cavity and becoming infected by micro-organisms. Decomposition follows, inflammation of the endometrium is the result, and the virulence of the infecting organism will be the determining factor as to the severity of the case.

Septic endometritis, on the other hand, is caused by streptococcic infection, and the entire endometrium is covered with a dirty, yellowish looking exudate, and not infrequently we find areas of necrosis. The resulting pathology from this condition is very frequently an inflammation of the Fallopian tubes, followed by infection of the ovaries, the whole affair at last becoming one of pyosalphix, ovarian abscess, or both. Should the infection travel as far as the fibrinated extremity of the tubes and should they thereby not become occluded, we get infection of the pelvic peritoneum, and later general peritonitis.

Should the infection not be highly virulent, the inflammatory condition may confine itself to the pelvic peritoneum. It is in this class of cases that we get the pelvic cellular tissue secondarily infected, and there is developed the condition known as parametritis. Let us bear in mind the anatomical fact, that the pelvic cellular tissue is extra-peritoneal, and forms a bed in which the pelvic organs, together with blood vessels, muscles, and all tissues forming the pelvic floor, are enclosed. All the pelvic cellular tissue is situated *above* the pelvic fascia which covers the levator ani muscle, and all the cellular tissue of the vulva and the ischio-rectal fossa lies below this fascia. The amount and relation of this cellular tissue in the pregnant state differ very materially from the non-pregnant state, and in our study of puerperal infection this can not be too carefully borne in mind. At the base of the broad ligaments it is very much increased. The ligaments are raised up to the brim by the growing uterus; in consequence of this, beneath the broad ligaments there is a triangular area on either side of the uterus, which is not covered by peritoneum, and as pregnancy advances, this area gradually becomes filled with new cellular tissue. Immediately contiguous to this new cellular tissue is a very large area situated behind Poupart's ligament. As the vesico-uterine pouch does not dip down into the pelvis as it did in the unimpregnated state, and the peritoneum does not dip into the lateral pelvis, we can readily see why the infection does not remain *pelvic* in the pregnant condition as it does in the non-pregnant. So also can we understand why so frequently in infection following delivery the cellular tissue *above the brim* becomes infected, and we have a large swelling behind Poupart's ligament. This altered arrangement of peritoneum and cellular tissue, which is brought about by pregnancy, very readily explains the position of some of the inflammatory swellings which occur during the puerperium, appearing as abdominal rather than pelvic, and which are not always so easy of diagnosis.

Pelvic cellulitis in the puerperal state must be a misnomer, for this parametritis is a result of a previous infection, and when we have the cellular tissue infected in this condition, it is always secondary to pelvic peritonitis.

The symptoms of puerperal infection will depend very largely upon the virulence of the infection, the type of tissue attacked and resistance offered by the individual to the absorption of the septic material. It is a very comforting belief to assume that the patient can and has infected herself; but the observations of men most competent to judge have been that this is not true except in certain instances. There may be an infecting coitus just previous to delivery, yet such a condition is by no means an auto-infection. More than fifty per cent. of prostitutes have latent gonorrhœa, and even in our best practice many honest women will be found in the same condition. These may, of course, infect themselves. There are many cases, also, in which there is a focus of pus in either one or both of the adnexa, who going to full term, may infect themselves independently of the method or manner of delivery; and yet such conditions must be, from the very nature of the case, rare indeed, because the result in such conditions is almost invariably abortion or sterility.

Upon a correct diagnosis depends effective treatment. We believe it to be of prime importance to the instituting of treatment in any given case of puerperal infection that an effort be made to differentiate the types of infection we have to deal with. All cases of puerperal infection can no more be brought to a successful issue by the same mode of procedure than all cases of appendectomy can be handled from the same standpoint. Therefore, when brought face to face with a case of infection, a decision must be reached as to its type.

It will be noted that in the gonococcic form the fever will have a regular run, which in the streptococcic form it will be found absent, and it will gradually decline to normal. The patient usually feels quite well and has a smooth puerperism during the first week. In other words, the onset is late. The symptoms will gradually subside and the patient feels quite comfortable, but for a considerable tenderness which continues in the region of the uterus and tubes. A parametritis is very rarely seen, though the tubes in many instances can be easily palpated. Whertheim has demonstrated, however, that the pelvic cellular tissue may be infected by the gonococcus. In streptococcic infection we have early onset, high fever in contrast to moderate fever; a rapid and oftentimes irregular pulse. In the gonococcic form we have a remission of fever in from thirty-six to forty-eight hours, which does not occur in the former.

In sapremia we have the history of retained placenta, foul-smelling lochia, etc. *There is no greater or more common error than*

*to suppose that in puerperal infection we always have a foul-smelling lachia.* In point of fact, in infections due to the streptococcus or the staphylococcus, the lochia in the vast majority of cases *will be free from odor.* In sapremia the uterus will be large, soft and more or less insensible to pressure, as against a small, firm and tender uterus in gonococcic infection. The essential point in the treatment of the gonococcic form is in the prophylaxis. We should begin at an early date in the case. If we have any doubt as to the previous morale of the tissues all discharges should be examined with the view to detecting latent gonorrhea. The simple history of the case taken by itself will be of little or no value, for increased urination, with burning sensations, together with an increased vaginal discharge, is such a common accompaniment of pregnancy as to make these symptoms, when taken in the abstract, absolutely valueless. The essentials are the microscopic findings. When a positive result has thus been obtained, the douche should not be neglected. Absolute rest *after delivery* must be enjoined and will be of much more value than the more active and energetic procedures.

In sapremic conditions the uterus must be freed of its abnormal contents. Nothing in point of safety and efficiency can take the place of the intelligent finger of the conscientious obstetrician. Of course, if this procedure be carried out in a half-hearted or apologetic manner, no good will follow. Therefore, anesthesia should be produced and a complete operative procedure instituted. For us to defer action in this condition until our patient becomes constitutionally and thoroughly infected is to place ourselves in an unjustifiable position. The *sine qua non* of success in the vast majority of these cases is an early recognition of the pathological condition and the early application of radical measures, which is truly the most conservative treatment. At this period it is simply necessary that we empty the uterus of all decomposing material. The curette, especially the sharp curette, should not be used. If the os has contracted it must be redilated, and if too much force be necessary to accomplish this with the finger then the Goodell's dilator may be used, keeping always in mind that the less traumatism produced the better. After emptying the uterine cavity, use large quantities of saline solution, swabbing out the entire cavity with fifty per cent. alcohol. If the procedure has been done early enough and thoroughly enough the result will be that the chills will cease, pulse and temperature will go to normal or thereabouts and remain. For years we have not used sublimate as an intra-uterine douche, for the more we see of it in this connection, the more convinced are we of its uselessness.

But the puerperal sepsis due to streptococcic infection, unlike gonorrheal or putrid, which tends to recovery, is rapidly fatal, or at best, seriously damages the pelvic organs to such a degree that our treatment must be governed not only by the *mortality* statistics, but by the *morbidity* as well. Curettage has given us a mortality that makes it prohibitive. This mode of treatment was very thoroughly gone over in a discussion of this subject before the Berlin Obstetrical Congress in 1891, and most of the authorities were opposed to this form of treatment. Fritsch called attention to the dangers of perforation and detachment of thrombi. He maintained that the *cervix* and not the uterus itself was the starting point of the infection in the great majority of cases. Garrigues states that he has never seen a case of recovery in which curettage has been resorted to after sepsis was well established.

It is a well-known fact that after all intra-uterine manipulations in puerperal sepsis, there is very frequently a temporary aggravation of the symptoms at least. This occurs not only after a curettage, but also after a digital exploration of the uterine cavity, or the giving of a simple intra-uterine douche.

Curetting is admissible only in cases in which we have evidence of retained products in the uterine cavity, and then it is to be accomplished by the intelligent finger of a conscientious obstetrician.

In 1898, a commission appointed by the American Gynecological Society reported that Marmorek's serum had been used in 101 cases of streptococcic puerperal fever with a mortality of thirty-three per cent. Pryor, of New York, found 257 cases in which Marmorek's serum was used, but in which no bacteriological diagnosis was made, which gave a mortality of sixteen per cent. Now, taking Williams', Kronig's and Franz's percentage statistics, we shall find that about one-quarter of them were streptococcic, and as the remaining seventy-five per cent. would, in all human probability, have gotten well without the serum, there were some forty deaths in about sixty-three cases—a mortality of sixty-three per cent. This is certainly a mortality which must be prohibitive as to the treatment used. In the vast majority of cases the infection begins near the external genitals, viz.: Tears in the perineum, vagina or cervix. If a thorough ocular examination more frequently took the place of the intra-uterine douche, we would be surprised to find how often the cavity of the uterus was *not* the invaded portal, but that the primary attack was nearer the external world, and traveled from there to the uterus, the Fallopian tubes, and lastly, the pelvic peritoneum. In not a few instances it reaches the pelvic peritoneum through the blood-vessels and lymphatics. All local treatment in the nature of intermittent washing and douching will be a waste of time. This is what Macharg did and had a death-rate of more than fifty per cent. The reason for this is not far to seek, for the pathogenic germs have passed in beyond the reach of such superficial treatment into the larger field of destructibility—*the general system.* Therefore, attack them in their first strong citadel. Make a broad incision into the posterior cul-de-sac. Do not make a vaginal puncture, which is an abomination unto all surgical procedures—but an incision free and generous. Through this all the fluids of the pelvis may be drained. In most cases we shall find much serum and lymph present, and in not a few some pus. Break down all false unions which may be found between the pelvic organs, and pack with iodoform gauze. I am well aware of the opposition from some quarters to iodoform gauze, yet we have not up to date been able to locate a case in which iodoform poisoning has occurred from the use of gauze thus employed, or used *within* the puerperal uterus. Therefore, we do not think that iodoform gauze thus used can be considered dangerous.

Not infrequently the pathogenic germs will be found in the serum of the cul-de-sac when they are absent in the uterine cavity, and this for the reason that the infection has not been through the uterus itself, but through some other portion of the genital canal. The patient's nourishment should be kept above par, if possible, but elimination must be attained to with equal regularity. Hypodermoclysis will be found of real value in all cases, increasing the action of the kidneys and skin, and increasing the volume of the pulse.

Only those drugs should be used that are either stimulant or tonic in action. Alcohol should be given with a free hand. The limit to its administration should be the general intoxication of the patient, and oftentimes an enormous amount will be consumed before the condition is produced. They must be liberally fed and given large quantities of water. An ice-bag over the abdomen will give great relief in dimishing the pain, and will tend to retard the extension of the pelvic peritonitis.

Much has been claimed of late for Ceede's ointment from some quarters. Personally, my experience with it has been very limited. "It is a soluble metallic silver made into a suitable ointment, which resembles very much the ordinary murcurial ointment. It is very rapidly absorbed and disseminated into the blood-current when applied to the skin." Roswell Park speaks very highly of it in general sepsis. However, it is one of the remedies which is still on trial, and one would hardly pin his faith to it alone in any given case of puerperal sepsis.

Hysterectomy for puerperal sepsis was most thoroughly discussed at the "Congress of Obstetrics and Gynecology." In Rome in September, 1902, Treub, of Amsterdam, expressed his opinion that hysterectomy for puerperal sepsis is very rarely indicated.

Tuffier, of Paris, gives to us the encouraging maxim; "To operate too early is criminal; to operate too late is *useless*." Either horn of the dilemma would seem to be equally undesirable.

Freund, of Berlin, would limit it to cases (1) of total or partial retention of placenta which resisted all efforts at removal in the ordinary way; (2) cases of abortion of criminal origin, when the treatment had not been aseptic, pyæmic symptoms, together with metritis, had developed. But in the latter period of puerperal sepsis, no matter whether it be of lymphangitic or of pyemic origin, extirpation should not be done.

Leopold, of Dresden, who read one of the principal papers, took quite an encouraging view of the subject.

Zweifel, of Leipzig, did not agree with Leopold as to the encouraging results obtained from hysterectomy in peritonitis of puerperal origin.

A. Pinad, of Paris, called attention to the fact that the advocates might be divided into two hostile camps, viz.: the obstetricians who were against it, and the gynecologists who were in favor of it.

Deaver, in a paper read before the American Surgical Association, makes use of the following language: "The results of hysterectomy in post-puerperal infection are more serious than extirpation for other infectious conditions. The constitutional disturbance must be the determining factor whether or not a hysterectomy shall be done; unfortunately, I regret to say that any operation is but too often contra-indicated for the above reason."

That hysterectomy is a justifiable procedure in the treatment of puerperal infection is admitted, but the indications are not always clear and well defined; therefore, it can not as yet be considered of general application even in selected cases.

The real treatment of puerperal infection should be begun before it has occurred. The general application of the antepartum and postpartum douche in normal cases should be absolutely *condemned and abolished*, and even in cases of forceps or manual delivery, if done with due precaution, we believe they should not be used, as they are by far a greater element of danger than of safety to the patient. There can be no question, it would seem, that the general use of rubber gloves in obstetrical work would bring a very much lower mortality and morbidity in puerperal infection. We believe this to be especially true in the case of the general practitioner, whose work naturally calls him into all kinds of surgical and infectious diseases.

To-night as we are discussing this most important subject can we not hear the final words of him who sleeps beneath the quiet shades of Auburn Hill, being echoed and re-echoed through the years of the departed century, "It is as a *lesson* rather than as a *reproach* that I call up the memory of these irreparable errors and wrongs. No tongue can tell the heart-breaking calamity they have caused; they have closed the eyes just opened upon a new world of love and happiness; they have bowed the strength of manhood into the very dust; they have cast the helplessness of infancy into the stranger's arms, or bequeathed it, with less cruelty, the death of its dying parent. There is no tone deep enough for regret and no voice loud enough for warning. The woman about to become a mother, or with her new-born infant upon her bosom, should be the object of trembling care and sympathy wherever she bears her tender burden, or stretches her aching limbs. The very outcast of the streets has pity upon her sister in degradation, when the seal of promised maternity is impressed upon her. The remorseless vengeance of the law brought down upon its victim by a machinery as sure as destiny, is arrested in its fall at a word which reveals her transient claim for mercy. The solemn prayer of the liturgy singles out her sorrows from the multiplied trials of life, to plead for her in her hour of peril. God forbid that any member of that profession to which she trusts her life, doubly precious at that eventful period, should hazard it negligently, unadvisedly or selfishly."—*Detroit Medical Journal.*

